# Comparison of three common whole blood platelet function tests for in vitro P2Y12 induced platelet inhibition

**DOI:** 10.1007/s11239-019-01971-1

**Published:** 2019-10-16

**Authors:** Joao D. Dias, Torben Pottgiesser, Jan Hartmann, Daniel Duerschmied, Christoph Bode, Hardean E. Achneck

**Affiliations:** 1 Haemonetics S.A., Signy, Switzerland; 2 Department of Cardiology and Angiology I, Faculty of Medicine, Heart Center Freiburg University, University of Freiburg, Freiburg, Germany; 3 Haemonetics Corporation, Braintree, USA

**Keywords:** Coagulation, Diagnostic, TEG^®^, Platelet function, Platelets

## Abstract

In the context of interventional cardiology, platelet function testing may identify patients treated with P2Y12-inhibitors at an increased risk of mortality, thrombosis and bleeding. Several whole blood point-of-care platelet function analyzers are available; however, inter-device differences have not been examined systematically. To compare three platelet function tests under standardized in vitro conditions. Healthy volunteer (n = 10) blood samples were spiked with increasing concentrations of ticagrelor (0–7500 ng/mL) and/or ASA (0–3280 ng/mL), measured on three platelet function analyzers (TEG^®^6s, Multiplate^®^, and VerifyNow^®^) and respective Effective Concentration (EC) levels EC10, EC50 and EC90 were calculated. Repeatability was assessed in a separate group of pooled blood samples (n = 10) spiked with ticagrelor at EC10, EC50 and EC90. ASA had no impact on ADP-activated channels for all three devices. TEG^®^6s was able to distinguish (p ≤ 0.05) between all ticagrelor EC zones; VerifyNow^®^ and Multiplate^®^ were able to distinguish between three and two zones, respectively. Multiplate^®^ showed the largest window between EC10 and EC90 (19–9153 ng/mL), followed by TEG^®^6s (144–2589 ng/mL), and VerifyNow^®^ (191–1100 ng/mL). Drug effect models distribution of disagreements were identified for TEG^®^6s (5.0%), VerifyNow^®^ (8.3%), and Multiplate^®^ (13.3%). TEG^®^6s showed the smallest average coefficient of variation between EC conditions (5.1%), followed by Multiplate^®^ (14.1%), and VerifyNow^®^ (17.7%). Linear models could be generated between TEG^®^6s and Multiplate^®^, but not VerifyNow^®^. Significant differences were found between whole blood point-of-care platelet function analyzers and the clinical impact of these differences needs to be further investigated.

**Electronic supplementary material:**

The online version of this article (10.1007/s11239-019-01971-1) contains supplementary material, which is available to authorized users.

## Highlights

- Platelet function devices have different measurement scales and so there is a lack of information comparing their performance under controlled conditions.
- Significant differences were found between three whole blood point-of-care platelet function analyzers.
- The TEG^®^6s analyzer was shown to have the highest degree of repeatability with the lowest level of disagreement between duplicate measurements.
- There is a need to develop and validate standardized cut-off values for these platelet function analyzers in order to find the optimal range of platelet reactivity.

## Introduction

Dual antiplatelet therapy (DAPT) consisting of acetylsalicylic acid (ASA) and a P2Y12 receptor blocker is recommended to reduce platelet reactivity and prevent thrombotic events after percutaneous coronary intervention (PCI) [1]. DAPT, however, is also associated with increased risk of bleeding as compared with treatment with a single platelet inhibitor and further increased with the potency of the combination partner [2]. Platelet function testing can be utilized to monitor therapy with P2Y12-inhibitors and identify low (LPR) and high (HPR) platelet reactivity during therapy [3]. In several consensus documents, cut-off values for LPR and HPR were extrapolated from the results of several clinical trials and linked to a higher risk of mortality and stent thrombosis (HPR) or an elevated risk for bleeding (LPR) [3, 4].

According to current revascularization guidelines (Class IIb recommendation), platelet function testing may be considered to de-escalate DAPT in patients with acute coronary syndrome; particularly in those deemed unsuitable for maintained potent platelet inhibition over 12 months [1, 5]. Furthermore, platelet function testing may be considered to guide the timing of cardiac surgery in patients who have recently received P2Y12 inhibitors [1, 6].

Several whole blood platelet function assessment devices that can be used in a near-patient set up are commercially available. Thromboelastography (TEG^®^) (Haemonetics, Braintree, MA, USA), Multiplate^®^ (Roche Diagnostics, Rotkreuz, Switzerland), PFA-100/PFA-200 (Siemens, Munich, Germany) and VerifyNow^®^ (Accumetrics, San Diego, CA, USA) systems are the most frequently studied. TEG^®^ PlateletMapping^®^ has been shown to be predictive of bleeding and thrombotic risk in patients undergoing PCI [7, 8], cardiac [9–11], and non-cardiac surgery [11, 12]; and the TEG^®^ DOAC cartridge can detect and classify direct oral anticoagulants with high specificity and sensitivity [13]. The clinical value of Multiplate^®^ in patients undergoing PCI or cardiac surgery [14–16], and VerifyNow^®^ in patients undergoing cardiac surgery [17, 18] has also been established.

Light transmission aggregometry (LTA) is a traditional platelet function test, but is poorly standardized and unlikely to be widely used in clinical practice [19]. Flow cytometry analyzes the functional status of platelets in vivo; specifically, it evaluates intracellular transduction. The efficacy of antiplatelet drugs can be monitored with flow cytometry through the intracellular quantification of Vasodilator Stimulated Phosphoprotein (VASP) phosphorylation [19].

Platelet function devices have different measurement scales and so there is a lack of information comparing their performance under controlled conditions. This is despite recommendations to perform platelet function testing to assess and manage bleeding and thrombosis, and the consensus-defined, uniform cut-offs for standardized platelet function assays [3]. In this study, we compared adenosine di-phosphate (ADP) tests (PlateletMapping^®^ ADP, Multiplate^®^-ADP and the VerifyNow^®^ P2Y12 Platelet Reactivity Test [P2Y12-PRU]) for their performance and correlation of results in response to antiplatelet inhibition in healthy volunteer blood samples spiked with ticagrelor and/or ASA.

## Materials and methods

### Study design

Blood samples were drawn from healthy donors, in accordance with ISO 17025 and Good Laboratory Practices (GLP). This study was conducted in two stages: the first study assessed the concentration dependent effects of ticagrelor and ASA on platelet function measurements obtained with the three analyzers (TEG^®^6s, Multiplate^®^, and VerifyNow^®^), while the second study evaluated the repeatability of measurement with each analyzer at fixed concentrations.

### Platelet function tests

#### TEG^®^6s

The TEG^®^6s hemostasis analyzer is a fully automated diagnostic instrument, employing a four channel cartridge system to quantify the viscoelastic properties of a whole blood clot from the enzymatic phase through to the fibrinolytic phase [20–22]. Four independent assays are conducted to provide a comprehensive overview of the patients’ coagulation status (citrated kaolin [CK], citrated kaolin with heparinase [CKH], citrated rapidTEG^®^ [CRT], and citrated functional fibrinogen [CFF]). The PlateletMapping^®^ cartridge allows selective activation of the P2Y12 receptor by ADP or through the thromboxane pathway using ASA.

#### Multiplate^®^

The Multiplate^®^ analyzer is a semi-automated point-of-care (POC) device using a five channel computerized system to perform multiple electrode impedance aggregometry in whole blood. Two independent sensor units detect impedance and automatically calculate area under the curve. Several activators are available, exploring different pathways of platelet activation in a similar process to LTA [23–25].

#### VerifyNow^®^

VerifyNow^®^ is a POC, turbidimetric-based optical detection device that provides a quantitative measure of platelet aggregation in whole blood [26]. This system provides two assays, each sensitive to targeted drugs. The aspirin assay is sensitive to ASA and utilizes arachidonic acid as an agonist [19]. The P2Y12 assay is sensitive to thienopyridines and uses ADP as an agonist and prostaglandin E1 (PGE1) as a suppressor of intracellular free calcium levels to reduce the nonspecific contribution of ADP binding to P2Y1 receptors [19]. Platelet aggregation results obtained from the VerifyNow^®^ P2Y12 assay are similar to those obtained with traditional LTA techniques [23, 27, 28].

### Study 1: concentration dependent effects of platelet aggregation inhibitors

Whole blood samples from 10 healthy volunteers were drawn using a three-step phlebotomy process to maintain the stability of thrombocytes. The maximum total blood draw per donor was 209 mL, and samples were collected using one of three blood tube types; 6 mL BD Vacutainer^®^ Lithium-Heparin tubes (TEG^®^6s), 3 mL Hirudin tubes (Multiplate^®^), or 2 mL Greiner Bio-One partial-fill VACUETTE^®^ tubes with 3.2% sodium-citrate (VerifyNow^®^). After each phlebotomy, the samples were equilibrated at room temperature for 30 min, before pooling the collected samples per patient for each blood tube type. The samples were then spiked (1:10) and incubated for 10 min before the start of analysis. Three blood draws were performed at 90-min intervals, and analyzed at the time of sampling. Samples for each run were spiked with six concentrations of ticagrelor (0–7500 ng/mL) in addition to ASA (0–3260 ng/mL); these doses were selected to represent prescribed drug regimens. This resulted in 18 drug combinations, generated in duplicate per donor for each of the three analyzers, for a total of 108 samples per donor. Details of the stock solution preparation and experiments to determine the appropriate final concentration of 1% DMSO (Supplementary Fig. 1) can be found in supplementary materials.

To analyze platelet function in the prepared samples, the PlateletMapping^®^-ADP test was performed for the TEG^®^6s, the ADP test for the Multiplate^®^ analyzer, and the P2Y12-PRU for the VerifyNow^®^ system. All assays were performed in line with the operational procedures detailed in the manual for each analyzer.

### Study 2: variability of the platelet function devices

Whole blood samples from 10 healthy volunteers were collected as described above; the maximum total blood draw per donor was 87 mL. This study compared the three devices to assess variability at Effective Concentration levels EC10, EC50, and EC90. Firstly, whole blood samples from 10 healthy donors without ticagrelor and ASA were tested with TEG^®^6s, Multiplate^®^ and VerifyNow^®^ systems in a single measurement. Samples were drawn from each donor and collected in the same three blood tube types as Study 1, with one blood tube of each type collected per donor and per phlebotomy. Secondly, pooled blood samples from 10 healthy donors were spiked with ticagrelor 10–15 min before measurement start, at EC10, EC50, and EC90 (Supplementary Table 1), and measured 10 times on each device. Working solutions of ticagrelor for spiking blood samples were prepared as described in the supplementary methods. Platelet function analysis of the prepared blood samples was conducted using the same tests per analyzer as detailed for Study 1.

### Statistical analysis

Descriptions of the visual analysis and modeling framework methods can be found in Supplementary Methods.

## Results

### Donor demographics

In total, 21 volunteers were enrolled in these studies; 11 volunteers participated in study 1, and ten volunteers in study 2. Overall, study participants (38% female/62% male) were healthy and currently receiving no medication, Caucasian, and had an average age of 39 years (range 19–57 years). In Study 1, 73% of the participants were male, with an average age of 45 years (range 20–57 years), and in Study 2, 50% of participants were male, with an average age of 32 years (range 19–49 years).

### Study 1: concentration dependent effects of platelet aggregation inhibitors

Drug effect curve models were successfully generated for each device (Supplementary Fig. 2). No ASA impact in the ADP-activated channels was observed, as confirmed by the p-values obtained by t-test for the three different regions of the dependent variable (Supplementary Table 2). There was no significant difference in the level of platelet aggregation inhibition across the three concentrations of ASA (p > 0.1). Similar results were seen across all three devices; therefore, ASA was not utilized in any further spiking experiments.

**Fig. 1:**
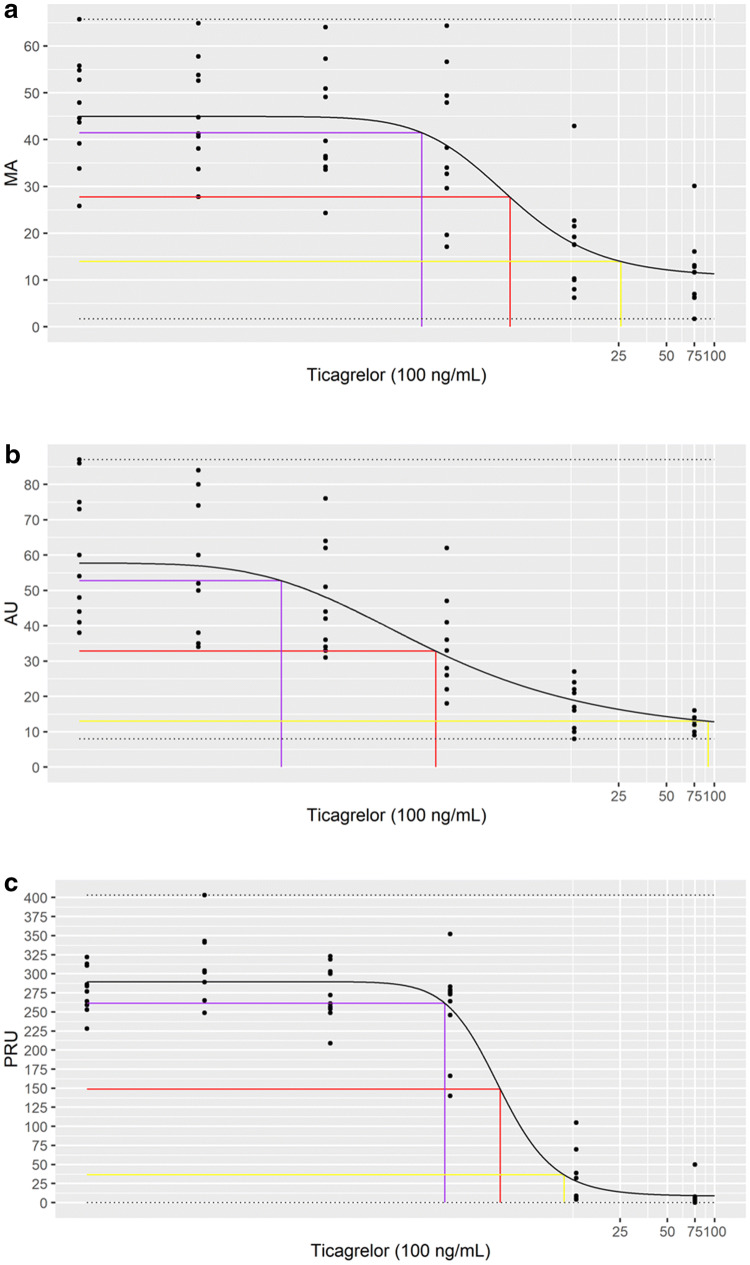
Effective dose analysis for ticagrelor. Graphs show fitted model plots for **a** TEG^®^, **b** Multiplate^®^, and **c** VerifyNow^®^ against log (ticagrelor). Black line represents the model curve, purple, red, and yellow lines represent EC10, EC50, and EC90, respectively. *MA* maximum amplitude, *AU* aggregation in aggregation units, *PRU* P2Y12 reaction units

The Effective Concentrations of ticagrelor (EC10, EC50, and EC90) were determined for each analyzer and the ability of each device to distinguish between four drug zones (< EC10, EC10–EC50, EC50–EC90, >EC90) was measured (Table 1 and Fig. 1). The TEG^®^6s was the only device able to distinguish between all four zones (p < 0.05). VerifyNow^®^ systems were able to distinguish between three drug zones, <EC10, EC50–EC90, and > EC90 (p ≤ 0.05), while Multiplate^®^ was able to distinguish between zones EC50–EC90 and > EC90 (p < 0.05) (Supplementary Table 3). Multiplate^®^ showed the largest window between EC10 and EC90 (19–9153 ng/mL), followed by TEG^®^6s (144–2589 ng/mL); VerifyNow^®^ showed the smallest window (191–1100 ng/mL) (Table 1).

**Table 1:** Effective dose calculations for ticagrelor

	EC10	EC50	EC90
TEG^®^6s -MA	144 [– 14, 301]	517 [24, 1010]	2589 [– 3734, 8912]
Multiplate^®^-AU	19 [– 6, 43]	176 [18, 334]	9153 [– 14511, 32818]
VerifyNow^®^-PRU	191 [124, 258]	431 [246, 615]	1100 [149, 2050]

Values given as ng/mL (with confidence interval)

*AU* aggregation in aggregation units, *EC* effective concentration, *MA* maximum amplitude, *PRU* P2Y12 reaction units

Distribution of disagreements were identified in the drug effect models for TEG^®^6s (5.0%), VerifyNow^®^ (8.3%), and Multiplate^®^ (13.3%).

### Study 2: variability of the platelet function devices

**Fig. 2:**
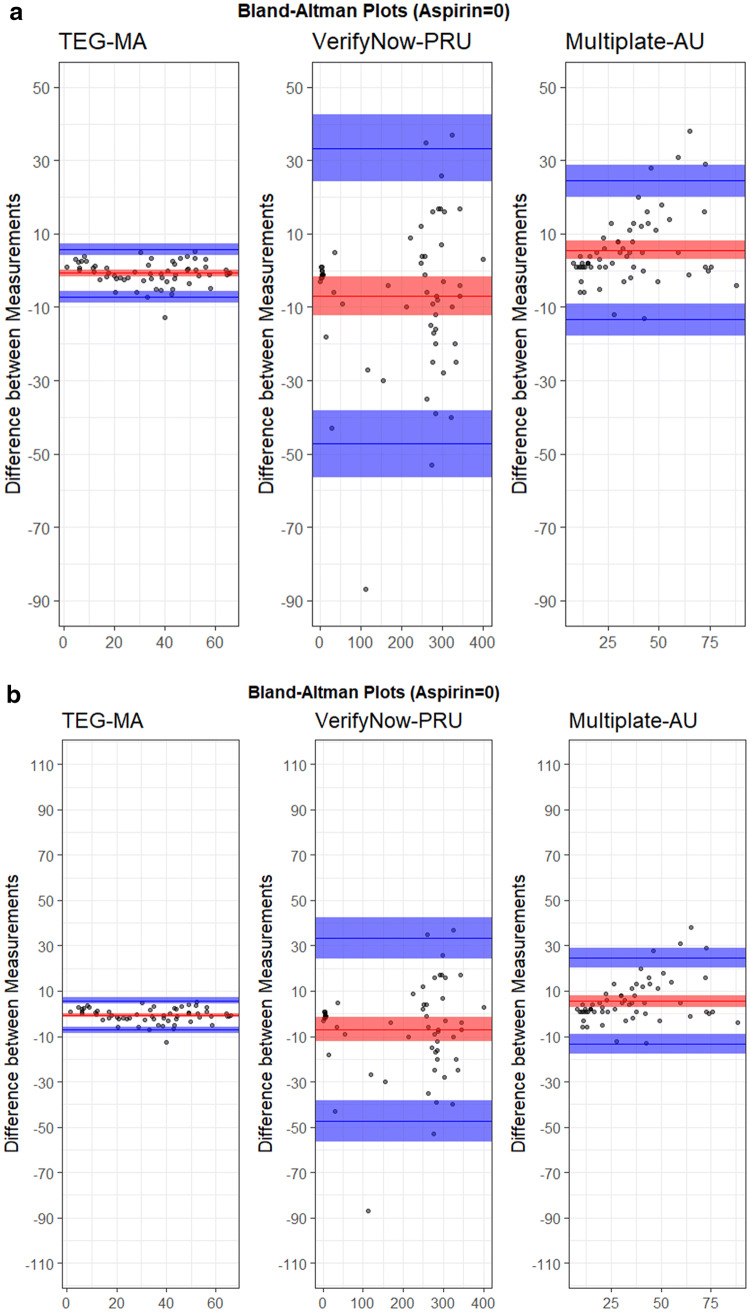
Device variability analysis for **a** unscaled data, and **b** data scaled to EC50. The plots show the difference between measurements versus the mean of measurements. Blue lines show minimum and maximum values with respective confidence intervals, red lines show the average value with confidence interval

The variability between measurements was evaluated for each device (Fig. 2). Mean (SD) of the measurements was − 0.72 (3.31) mm for TEG^®^6s, 5.55 (9.68) AU for Multiplate^®^, and − 6.97 (20.59) PRU for VerifyNow^®^. TEG^®^6s showed the smallest average coefficient of variation between EC conditions (5.1%), followed by Multiplate^®^ (14.1%), and VerifyNow^®^ (17.7%) (Table 2). The data was also scaled based on the device-specific EC50 window and TEG^®^6s still showed the smallest coefficient of variability (50.6%), followed by VerifyNow^®^ (61.2%), and Multiplate^®^ (72.8%) (Supplementary Table 4). Based on the device performance data agreement, linear models could be generated between TEG^®^6s and Multiplate^®^, but not VerifyNow^®^ (Supplementary Table 5).

**Table 2:** Summary statistics for variability assessment of each device (data not scaled)

	Mean	Median	SD	CV (%)	MAD	LOO Mean	Average of CV (%)
EC10_TEG^®^6s	49.0	49.4	1.5	3.0	1.7	2.8	5.1
EC50_TEG^®^6s	45.1	45.2	3.1	6.8	1.9	7.4	
EC90_TEG^®^6s	10.6	10.7	0.6	5.6	0.4	1.4	
EC10_Multiplate^®^	22.5	22.0	3.0	13.3	3.0	5.0	14.1
EC50_Multiplate^®^	17.1	17.0	1.9	10.8	3.0	3.2	
EC90_Multiplate^®^	15.4	15.5	2.8	18.2	1.5	5.1	
EC10_VerifyNow^®^	234.6	237.5	21.7	9.2	27.4	40.7	17.7
EC50_VerifyNow^®^	156.6	156.5	33.0	21.1	46.0	55.1	
EC90_VerifyNow^®^	6.6	7.0	1.5	22.8	1.5	2.9	

*CV* coefficient of variability, *EC* effective concentration, *LOO* leave-one-out, *MAD* mean absolute deviation, *SD* standard deviation

## Discussion

For this comparison, an in vitro model of thienopyridine sensitive platelet function inhibition was utilized. Whole blood samples spiked with ticagrelor at doses equivalent to prescribed drug regimens, were tested for platelet function inhibition using three commercially available platelet function devices. The results presented here demonstrate that the TEG^®^6s and Multiplate^®^ devices have consistent, interchangeable results. In contrast, high variability was seen between results from the TEG^®^6s and Multiplate^®^ devices with the VerifyNow^®^ device, meaning these results could not be correlated using a linear model. The TEG^®^6s analyzer was shown to have the highest degree of repeatability with the lowest level of disagreement between duplicate measurements. Multiplate^®^ had the highest average variance between repeat measurements, and VerifyNow^®^ had the lowest level of repeatability under device normalized conditions.

This is the first study comparing TEG^®^6s, Multiplate^®^ and VerifyNow^®^ under standardized conditions and at device normalized drug concentrations. By normalizing the study conditions to the device, we have been able to perform a clinically relevant comparison between devices despite the varying scales and movement patterns. VerifyNow^®^ was found to have the narrowest window between EC10 and EC90 (191 ng/mL and 1100 ng/mL), which is equivalent to a 60 mg ticagrelor pill (EC10) and close to the normal maximum blood concentration for 180 mg ticagrelor (a dose of two 90 mg pills) (EC90). Furthermore, we have shown that the VerifyNow^®^ device had a high coefficient of variation (> 20%), particularly in the important drug ranges of EC50 and EC90. A potential benefit of this narrowest window performance to ticagrelor is a lower influence of measurement timing relative to when the last ticagrelor dose was taken. This also would suggest that TEG^®^6s and Multiplate^®^ may be more sensitive to the peak and trough effects observed with pharmacodynamic measurements in the setting of ticagrelor use. Several studies using the VerifyNow^®^ device to guide therapy in clinical settings have shown no significant improvement in patient outcomes [29–31]. This may be due to study design; additionally, it has previously been reported that VerifyNow^®^ may overestimate the therapeutic response to clopidogrel in some individuals, potentially due to the utilization of PGE1 in addition to ADP [32]. This is in line with the results of our study, where low interchangeability of VerifyNow^®^ results were seen with TEG^®^6s and Multiplate^®^ results, and is further confirmed by a previous study noting poor agreement between VerifyNow^®^ and Multiplate^®^ [33].

The ability of TEG^®^ PlateletMapping^®^ to identify statistically significant platelet inhibition following antiplatelet therapy has previously been described [34]. Overestimation of platelet aggregation inhibition by ASA has been reported for VerifyNow^®^ and Multiplate^®^ [35]. However, in our study, there was no interference from ASA on ADP-induced platelet aggregation at routine prescribed doses for DAPT; this is in line with other studies using multiple electrode aggregometry to evaluate dual platelet inhibition [36].

Several studies have found that the platelet function analyzers assessed here can give variable results when defined cut-off values are used to predict clinical outcomes [37, 38]. Published cut-off data for TEG^®^6s and Multiplate^®^ were found to be interchangeable during this study, but the cut-off values for VerifyNow^®^ were not. The proposed cutoff values described in this study were selected from measurements in sufficiently large patient populations, meaning that inter-patient variability and test variability are already present and accounted for in the cut-off selection analysis. Contrary to the results described here, a study comparing TEG^®^ with multiple electrode impedance aggregometry reported that while the TEG^®^ PlateletMapping^®^ assay was predictive of bleeding, Multiplate^®^-ADP had a limited ability to predict transfusion requirements in cardiovascular surgery [10]. This disagreement with our results could potentially be explained by differences in the coefficient of variation between these two devices. Indeed, when we conducted the assessment using device-scaled conditions, Multiplate^®^ displayed the highest coefficient of variability. Despite the collaborative analysis attempt towards clinical validation of cut-off points for platelet function testing in a large sample of patients undergoing PCI [3], our results clearly identify the need to develop standardized cut-off values for these platelet function analyzers in order to find the optimal range of platelet reactivity discriminating bleeding and ischemia with the lowest rates of adverse events as proposed by the consensus document [3].

To our knowledge, this is the first time an ex vivo model has been described to test platelet function devices at clinically relevant concentrations of P2Y12 inhibitors in human whole blood. A previous study used TEG^®^ and Multiplate^®^ to assess the efficacy of hemostatic agents to improve hemostasis in vitro in ticagrelor-spiked blood samples; however, the ticagrelor concentrations used were not equivalent to prescribed dose regimens [39].

This study had several limitations, including that this was an in vitro experiment using blood samples from healthy human volunteers who do not represent the target population; however, this allowed for comparison of the platelet function analyzers under clearly defined, well-controlled conditions. Another limitation is that there is still some overlap between distributions of TEG^®^6s measurements over different ticagrelor concentrations due to the small sample size and DMSO usage. Once more data is collected, the hierarchical variability of measurements for the various concentrations of ticagrelor are expected to converge. Although it is difficult to draw clinical conclusions from this in vitro study, the data presented demonstrates that the TEG^®^6s device produces consistent, interchangeable results and that it is therefore a useful tool for monitoring platelet function. Confirming the clinical utility of the TEG^®^6s device is an important future direction and this could include in vivo testing and additional comparisons with other platelet function tests such as VASP. Another area of interest to explore in future studies, would be to examine how the therapeutic window and cut-offs for each assay relate to thrombosis and bleeding risk. This analysis was not carried out in the current study, as patient data on bleeding and thrombosis was not available. However this analysis would be beneficial for future studies in order to demonstrate the clinical utility of these assays in monitoring the effect of drugs in reducing thrombosis risk. Other potential limitations of the study include manipulation of samples in vitro that may introduce additional variability not present in unaltered blood samples from patients taking antiplatelet therapy, and the addition of DMSO that may have had an impact on platelet function and may adversely affect repeatability. However, our overall results were in line with previously published work where > 1000 fold ticagrelor dosing was required due to the reduced solubility when not including DMSO [39]. For this study, we were able to use dosing equivalent to the prescribed dosing regimen for these drugs.

There are several platelet aggregation inhibitors used frequently for DAPT; however, ticagrelor was the only platelet aggregation inhibitor used in these experiments, as ASA was found not to interfere with the inhibition of platelet aggregation. As ticagrelor is a reversible P2Y12 receptor inhibitor, the results cannot be extended to the thienopyridine class of P2Y12 inhibitors including clopidogrel and prasugrel.

## Conclusion

In this study, results from the analyzers TEG^®^6s and Multiplate^®^ could be correlated but not with those from VerifyNow^®^. Significant differences of repeatability and consistency of results were found between the different analyzers and the clinical impact of these differences in patient outcomes need to be further investigated in clinical trials.

Despite the collaborative analysis attempt towards clinical validation of standardized cut-off points for platelet function testing [3], there is a need to systematically develop standardized cut-off values for these platelet function analyzers in order to find the optimal range of platelet reactivity with validation in a randomized controlled trial enabling personalized antiplatelet therapy.

## Electronic Supplementary Material

Below is the link to the electronic supplementary material

Supplementary material 1 (DOCX 197 kb)
