# Supplementary material for: Comparison of three common whole blood platelet function tests for in vitro P2Y12 induced platelet inhibition
Source: J Thromb Thrombolysis. 2019 Oct 16;50(1):135–43. doi: 10.1007/s11239-019-01971-1 (PMC7293977; doi:10.1007/s11239-019-01971-1)
Supplement: Supplementary file 1 — Supplementary material 1 (DOCX 197 kb) [file 11239_2019_1971_MOESM1_ESM.docx]

# Supplementary materials

**Methods**

*Stock solution preparation*

ASA (Cayman Chemical, MI, USA), was dissolved in phosphate-buffered saline (PBS) to prepare a 5mg/mL stock solution. Serial dilutions in PBS were performed to obtain working solutions, and added to the samples. 5 mg Ticagrelor (Cayman Chemical, MI, USA) was reconstituted in 250μL dimethyl sulfoxide (DMSO) to prepare a 20 mg/mL stock solution. Serial dilutions in 10% DMSO were performed to obtain the working solutions.

*DMSO concentration*

Experiments were conducted to determine the appropriate final concentration of DMSO for this study. There was no difference in platelet aggregation inhibition with ticagrelor concentrations of 0 ng/mL or 444 ng/mL when dissolved in >1.42% DMSO (Supplementary Figure 1a). At a DMSO concentration of <0.5%, there was a significant reduction in ticagrelor-induced platelet aggregation inhibition due to poor solubility, which became more pronounced with higher doses of ticagrelor (Supplementary Figure 1b). Up to a 20% reduction in platelet aggregation inhibition was seen when using DMSO at 0.5% compared with DMSO at 1%; therefore, a 1% concentration was used to balance the highest level of solubility with the lowest impact on platelet function.

*Statistical analysis*

For every device, visual analysis was used to provide the initial assessment between the drug concentration and the platelet function measurements. The relationship was further studied in an appropriate modeling framework informed by the visual analysis, including three or four parameter Emax models. The performance of the models was assessed using model diagnostics and goodness of fit measures, and the final modeling framework was selected. In the presence of strong evidence of a non-monotonic relationship, a semi-parametric modeling framework was explored. In particular, the models based on B-splines were investigated as candidates. Upon selection of the final model, the parameters such as EC50 were estimated.

A four parametric log-logistic model was used for TEG®-MA and VerifyNow®-PRU parameters; this was also used for Multiplate®-AU with the restricted asymptotic to minimum observed value for multiple electrode impedance aggregometry-AU. Disagreements were not removed from the analysis.

Bland-Altman plots were used for the visual analysis of the two replicates for the given drug concentrations. The distributions of the relative errors were estimated and reported for each device. Statistical assessment of the distribution of the measurement and coefficient of variation was conducted and reported for each device. Summary statistics were calculated with scaled and unscaled data.

The effective dose analysis was conducted using only the first replicate from Study 1, with no ASA added to samples. Linear models were used for the comparison of effective dose, and 95% confidence intervals for the cutoff values were rounded.

**Supplementary Figures**

**Supplementary Figure 1:** Concentration dependent effects of DMSO on ticagrelor-induced platelet aggregation inhibition

(a)

DMSO, dimethyl sulfoxide; MA, maximum amplitude

(b)

DMSO, dimethyl sulfoxide; MA, maximum amplitude

**Supplementary Figure 2:** Model fit visualization of concentration dependent effects of ASA and ticagrelor for (a) TEG®6s, (b) Multiplate®, and (c) VerifyNow®

(a)


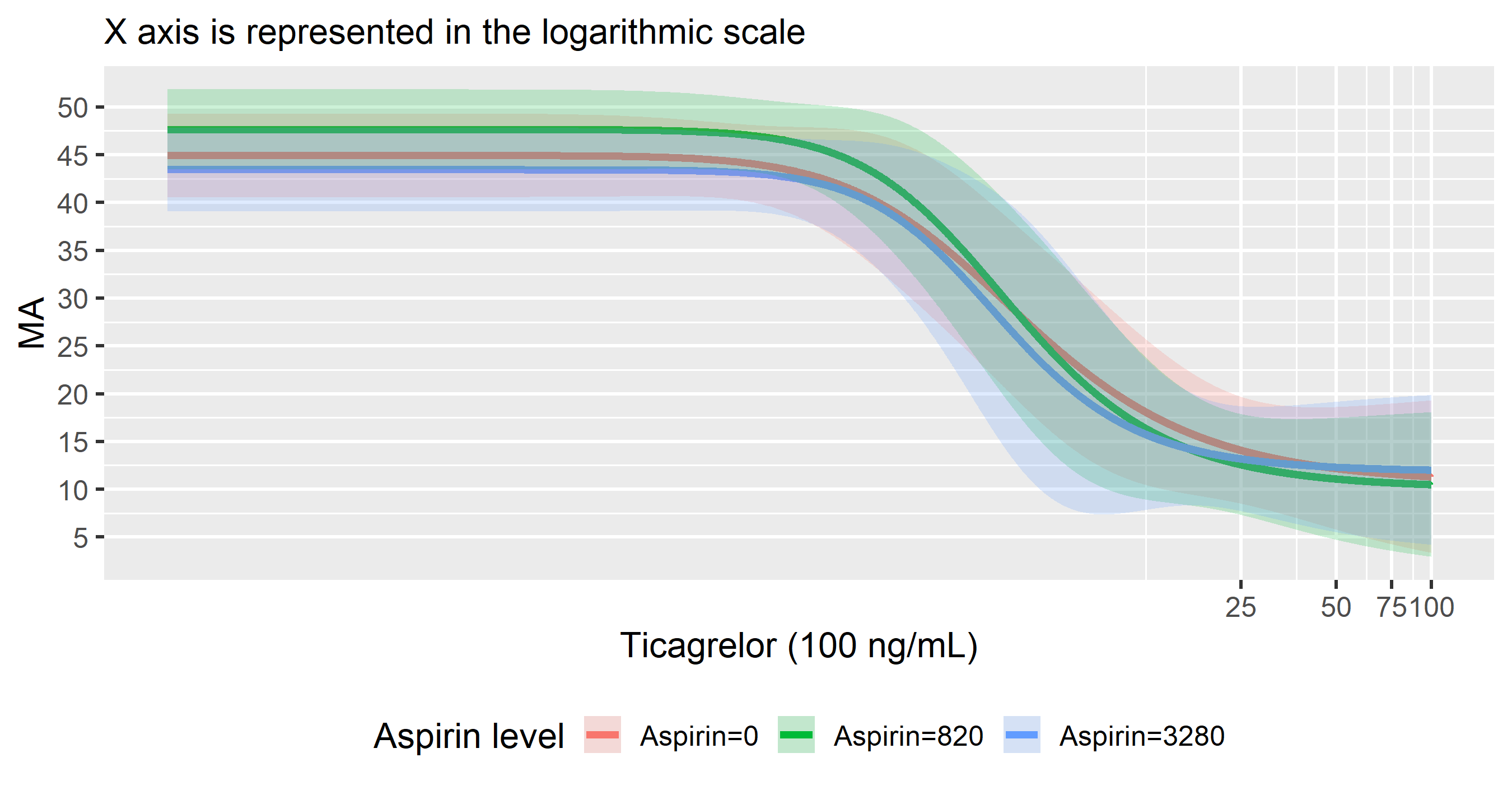


MA, maximum amplitude

(b)


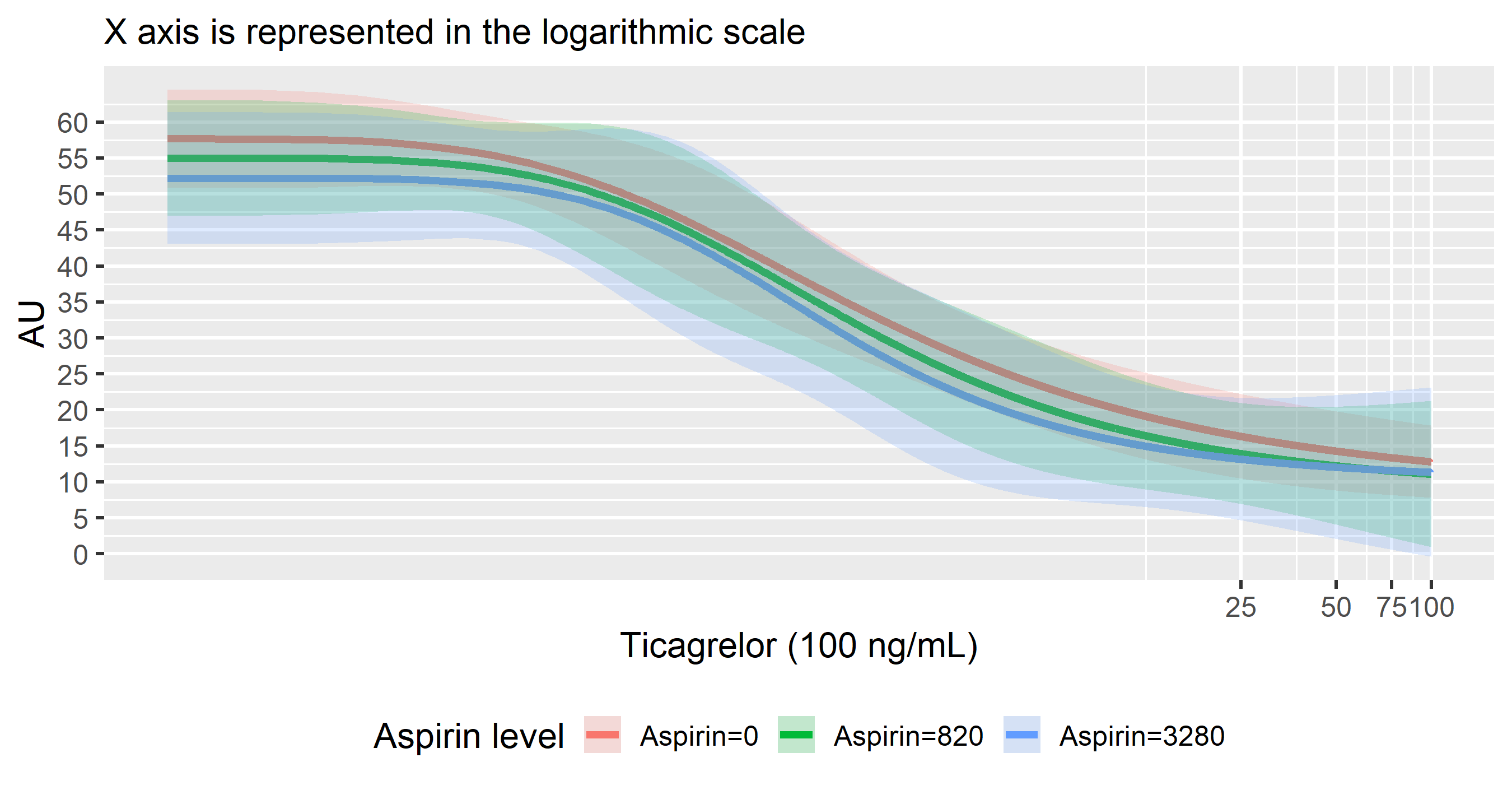


AU, aggregation in aggregation units

(c)


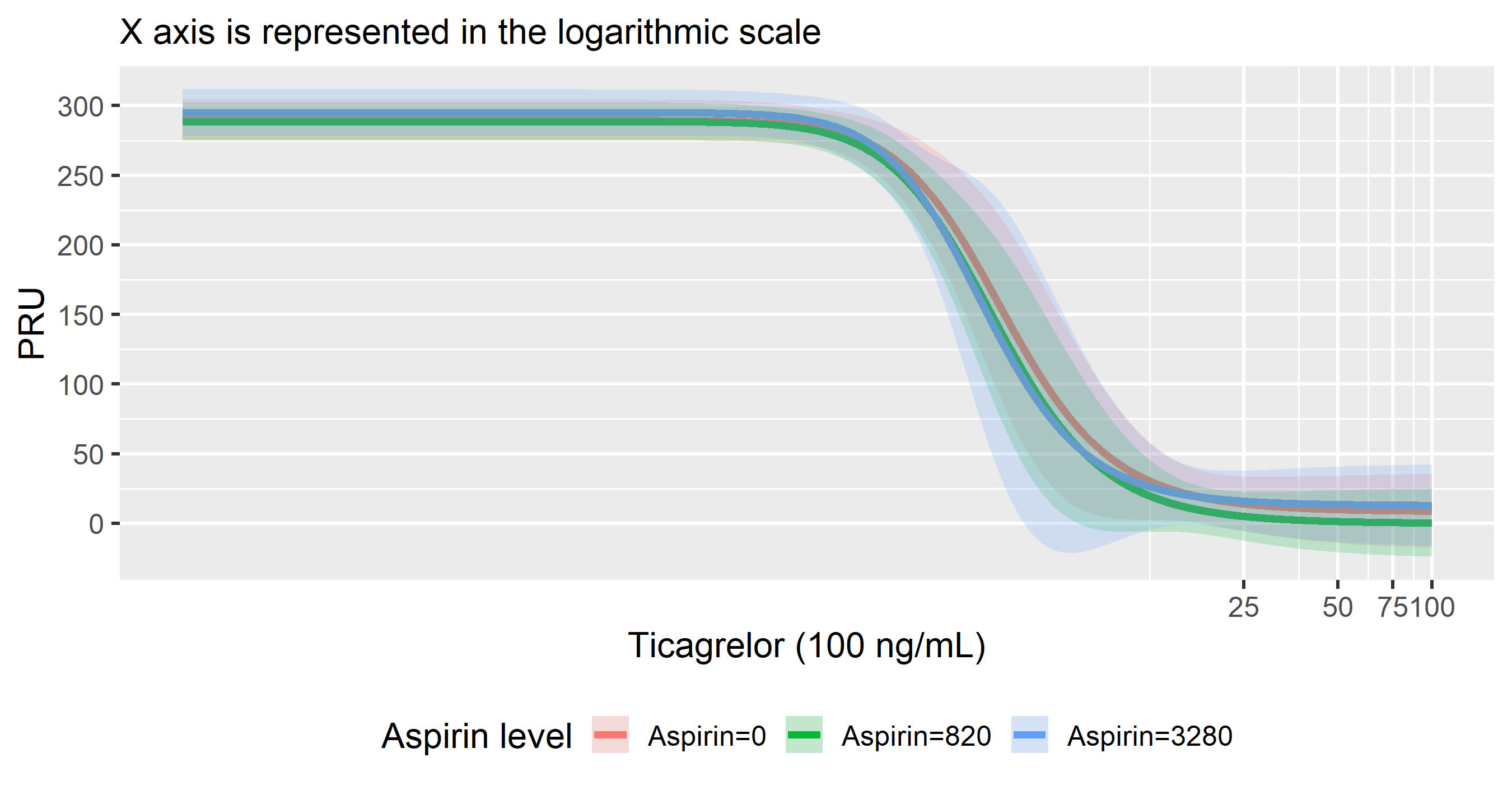


PRU, P2Y12 reaction units

**Supplementary Tables**

**Supplementary Table 1:** Ticagrelor volume required to establish Effective Concentration levels for the TEG®6s, Multiplate® and VerifyNow® systems. Values given as ng/mL

| **Based on Average Results** | **TEG®6s** | **Multiplate®** | **VerifyNow®** |
| --- | --- | --- | --- |
| **EC10** | 27 | 140 | 198 |
| **z** |  |  |  |
| **EC50** | 229 | 509 | 468 |
| **EC90** | 7576* | 2607 | 1269 |

*The maximum ticagrelor concentration in the blood sample without precipitation is 7500 ng/mL; this concentration will be used for spiking experiments

**Supplementary Table 2:** P-values from assessment of concentration-dependent effects of acetylsalicylic acid (ASA) on inhibition of platelet aggregation

|  | **ASA concentration** | | |
| --- | --- | --- | --- |
|  | **0 ng/mL vs. 820 ng/mL** | **0 ng/mL vs. 3280 ng/mL** | **820 ng/mL vs. 3290 ng/mL** |
| **TEG®6s** | | | |
| 0–10% | 0.887 | 0.655 | 0.887 |
| 10–90% | 0.902 | 0.700 | 0.902 |
| 90–100% | 0.796 | 0.418 | 0.796 |
| **Multiplate®** |  |  |  |
| 0–10% | 0.855 | 0.568 | 0.855 |
| 10–90% | 0.732 | 0.282 | 0.732 |
| 90–100% | 0.874 | 0.618 | 0.874 |
| **VerifyNow®** |  |  |  |
| 0–10% | 0.887 | 0.655 | 0.887 |
| 10–90% | 0.902 | 0.700 | 0.902 |
| 90–100% | 0.796 | 0.418 | 0.796 |

**Supplementary Table 3:** Model summary statistics of ticagrelor Effective Concentration analysis to determine the ability of each device to distinguish between four drug zones (<EC10, EC10–EC50, EC50–EC90, >EC90)

|  | **Estimate** | **Standard error** | **t-value** | **p-value** |
| --- | --- | --- | --- | --- |
| **Fitted model: TEG®6s-MA ~ log(ticagrelor)** | | | | |
| <EC10 | 9.5770 | 4.6308 | 2.0681 | p≤0.05* |
| EC10–EC50 | 10.4988 | 5.3512 | 1.9620 | p≤0.05* |
| EC50–EC90 | 44.9392 | 2.1845 | 20.5715 | p≤0.001* |
| >EC90 | 2.7134 | 0.2067 | 13.1297 | p≤0.001* |
| **Fitted model: Multiplate®-AU ~ log(ticagrelor)** | | | | |
| <EC10 | 2.7125 | 2.1998 | 1.2331 | 0.222 |
| EC10–EC50 | -4.5520 | 38.5017 | -0.1182 | 0.906 |
| EC50–EC90 | 58.8421 | 4.5861 | 12.8304 | p≤0.001* |
| >EC90 | 2.6112 | 1.2639 | 2.0661 | p≤0.05* |
| **Fitted model: VerifyNow®-PRU ~ log(ticagrelor)** | | | | |
| <EC10 | 15.2855 | 3.6985 | 4.1329 | p≤0.001* |
| EC10–EC50 | 8.3741 | 13.8514 | 0.6046 | 0.547 |
| EC50–EC90 | 289.5912 | 7.4515 | 38.8636 | p≤0.001* |
| >EC90 | 2.6340 | 0.0928 | 28.3908 | p≤0.001* |

*statistically significant (p≤0.05)

**Supplementary Table 4:** Summary statistics for variability assessment of each device (scaled data)

|  | **Mean** | **Median** | **SD** | **CV (%)** | **MAD** | **LOO Mean** | **Sum of Squares** | **Average**  **Of CV (%)** |
| --- | --- | --- | --- | --- | --- | --- | --- | --- |
| **EC10_TEG®6s** | 54.5 | 63.8 | 31.3 | 57.4 | 1.7 | 60.5 | 8811.2 | 50.6 |
| **EC50_TEG®6s** | 59.2 | 60.2 | 27.3 | 46.1 | 1.9 | 65.8 | 6698.2 |  |
| **EC90_TEG®6s** | 55.5 | 59.1 | 26.8 | 48.4 | 0.4 | 61.6 | 6479.3 |  |
| **EC10_Multiplate®** | 50.0 | 44.4 | 33.2 | 66.5 | 3.0 | 55.6 | 9938.3 | 72.8 |
| **EC50_Multiplate®** | 42.0 | 40.0 | 37.1 | 88.2 | 3.0 | 64.4 | 12360.0 |  |
| **EC90_Multiplate®** | 48.9 | 50.0 | 31.1 | 63.6 | 1.5 | 56.8 | 8691.4 |  |
| **EC10_VerifyNow®** | 60.0 | 64.8 | 35.5 | 59.2 | 27.4 | 66.7 | 11358.2 | 61.2 |
| **EC50_VerifyNow®** | 55.7 | 55.6 | 37.1 | 66.6 | 46.0 | 61.9 | 12408.0 |  |
| **EC90_VerifyNow®** | 65.0 | 75.0 | 37.6 | 57.9 | 1.5 | 72.2 | 12750.0 |  |

CV, coefficient of variability; EC, effective concentration; LOO, leave-one-out; MAD, mean absolute deviation; SD, standard deviation

**Supplementary Table 5:** Prediction of previously published cut-off values by TEG®6s, and Multiplate® (confidence intervals included); VerifyNow® results were not able to be correlated

|  | **Cut-off Values** | **by TEG®6s** | **by Multiplate®** |
| --- | --- | --- | --- |
| **TEG®6s Low** | 31 | - | 27.37 [25-30] |
| **TEG®6s High** | 47 | - | 41.9 [39-44] |
| **Multiplate® Low** | 19 | 33.45 [31-36] | - |
| **Multiplate® High** | 46 | 45.44 [42-49] | - |
